# Determinants of institutional birth among women in rural Nepal: a mixed-methods cross-sectional study

**DOI:** 10.1186/s12884-016-1022-9

**Published:** 2016-08-27

**Authors:** Sheela Maru, Sindhya Rajeev, Richa Pokhrel, Agya Poudyal, Pooja Mehta, Deepak Bista, Lynn Borgatta, Duncan Maru

**Affiliations:** 1 Bayalpata Hospital, Possible, Sanfebagar-10, Achham Nepal; 2 Department of Obstetrics and Gynecology, Boston Medical Center, Boston, MA USA; 3 Department of Obstetrics and Gynecology, Boston University School of Medicine, Boston, MA USA; 4 Department of Medicine, Division of Women’s Health, Brigham and Women’s Hospital, Boston, MA USA; 5 Bellevue Hospital Center, Ronald O. Perelman Department of Emergency Medicine, New York University School of Medicine, New York, NY USA; 6 City of Berkeley, Berkeley, CA USA; 7 Faculty of Health and Life Sciences, Department of Biological and Life Sciences, Oxford Brookes University, Oxford, United Kingdom; 8 Department of Medicine, Division of Global Health Equity, Brigham and Women’s Hospital, Boston, MA USA; 9 Department of Medicine, Division of General Pediatrics, Boston Children’s Hospital, Boston, MA USA; 10 Department of Medicine, Harvard Medical School, Boston, MA USA

**Keywords:** Institutional birth rate, Maternal mortality, Skilled birth attendants, Women’s health, Global health, Implementation research, Nepal

## Abstract

**Background:**

Encouraging institutional birth is an important component of reducing maternal mortality in low-resource settings. This study aims to identify and understand the determinants of persistently low institutional birth in rural Nepal, with the goal of informing future interventions to reduce high rates of maternal mortality.

**Methods:**

Postpartum women giving birth in the catchment area population of a district-level hospital in the Far-Western Development Region of Nepal were invited to complete a cross-sectional survey in 2012 about their recent birth experience. Quantitative and qualitative methods were used to determine the institutional birth rate, social and demographic predictors of institutional birth, and barriers to institutional birth.

**Results:**

The institutional birth rate for the hospital’s catchment area population was calculated to be 0.30 (54 home births, 23 facility births). Institutional birth was more likely as age decreased (ORs in the range of 0.20–0.28) and as income increased (ORs in the range of 1.38–1.45). Institutional birth among women who owned land was less likely (OR = 0.82 [0.71, 0.92]). Ninety percent of participants in the institutional birth group identified safety and good care as the most important factors determining location of birth, whereas 60 % of participants in the home birth group reported distance from hospital as a key determinant of location of birth. Qualitative analysis elucidated the importance of social support, financial resources, birth planning, awareness of services, perception of safety, and referral capacity in achieving an institutional birth.

**Conclusion:**

Age, income, and land ownership, but not patient preference, were key predictors of institutional birth. Most women believed that birth at the hospital was safer regardless of where they gave birth. Future interventions to increase rates of institutional birth should address structural barriers including differences in socioeconomic status, social support, transportation resources, and birth preparedness.

**Electronic supplementary material:**

The online version of this article (doi:10.1186/s12884-016-1022-9) contains supplementary material, which is available to authorized users.

## Background

### Introduction

The maternal mortality ratio in Nepal is estimated at 281 per 100,000; approximately half of these cases are attributed to postpartum hemorrhage [1]. Given that active management of the third stage of labor minimizes obstetric morbidity and mortality in low-resource settings, increasing the rate of births that take place in a medical facility is a key approach to reducing maternal mortality [2–4]. Despite efforts in Nepal to increase rates of institutional birth, 35 % of births nation-wide take place in a medical facility and in the impoverished Far-Western Development Region where this study takes place, that percentage is only 29 %. In Nepal, only 5 % of the estimated need for emergency obstetric care is met, and only 0.7 % of births are by cesarean section compared to the United Nations target of 5–15 % [5].

In a literature review of the determinants of birth service use in the Global South, maternal age, parity, education, household wealth, and urban residence were positively associated with service usage [6–9]. In a key study done by the Nepal Safe Motherhood Program in five districts in Nepal, 96 women were interviewed about their decision to give birth at an emergency obstetric care facility. When asked about why they chose a particular emergency obstetric care facility, availability of the full range of obstetric services was an important determinant (35 % of mothers). Of note, 2 % of the mothers reported making a decision as to where to give birth themselves, while 83 % of mothers reported that male members of the family made the decisions [10]. Other published works from Nepal confirm that family members influence the decision to give birth at home [11, 12].

### Significance

Specific barriers to safe institutional birth can be understood within the framework of the Three Delays Model: first, delay in the decision to seek care, second, delay in arrival at a health facility, and third, delay in the provision of appropriate care at the healthcare facility [13, 14]. The objective of this study is to understand which factors determine a woman’s decision to have an institutional birth in rural Nepal. Study findings will inform priorities in scale up to comprehensive emergency obstetric care, quality improvement strategies, and the creation of more responsive community-based and hospital-based antenatal, obstetric, and postpartum programming in rural Nepal. These findings may also be useful to the development of comprehensive emergency obstetric care programs in culturally similar, low-resource settings.

## Methods

### Study design

Data for this study was obtained from the Pre-Surgical Expansion Survey (Additional file 1) administered for 3 months in 2012 in the area around Bayalpata Hospital. The sample was comprised of 1) postpartum women in the hospital’s catchment area population who had given birth at home, and 2) postpartum women who had given birth at the hospital or at a village clinic. Written informed consent, either by signature or thumbprint on a consent form, was obtained for both the quantitative survey and the qualitative interview. This is the first part of a larger study to explore how decision-making changes after expansion of obstetric services at a district hospital. The data from the second survey period (Post-Surgical Expansion Survey) will be compared to the Pre-Surgical Expansion Survey data and presented in a subsequent publication.

### Setting

Bayalpata Hospital is located in Achham, a rural district in the Far-Western Development Region of Nepal. It is a district-level hospital run through a private-public partnership between the Ministry of Health of Nepal and the Nepali non-profit organization *Possible*. Services are provided to patients of the hospital free of charge. These services include birth services, laboratory diagnostics, an on-site pharmacy, and an operating theatre. The Community Health Program includes counseling and tracking of pregnant women by Community Health Workers (CHWs; known locally in Nepal as Female Community Health Volunteers). The CHWs’ coverage area, at the time of research, included seven village clusters (known locally in Nepal as Village Development Committees) around the hospital that contributed a large portion of patients to Bayalpata Hospital and defined a catchment area population of approximately 17,000 people for this study. At the time of the survey, the hospital’s ambulance service charged a distance-based fee, while the hospital provided Basic Emergency Obstetrics Care, with no capacity for cesarean sections or blood transfusion.

### Selection of participants

Study parameters included a convenience sampling of nearly all women who gave birth in the home setting as well as an emergency obstetric care facility over a 3 month period. Taking the findings of the Nepal Safe Motherhood study into account, which found that only 2 % of the mothers make the decision regarding where to give birth themselves, the study team sought to include a subset of male family members. However, because the survey’s male sample size was too small to analyze trends, this group was ultimately excluded. Therefore the final inclusion criteria included only post-partum women in the catchment area population who had given birth in the previous 6 weeks.

Power was calculated for a planned evaluation of the Pre- and Post- Surgical Expansion Survey data. Using an algorithm based on the Wald test, with power set at 0.80, statistical significance set at 0.05, and a baseline institutional birth rate of 20 %, the study was powered to detect a 150 % increase (i.e., to 30 %) in institutional births after the implementation of comprehensive emergency obstetric services. This power calculation required a total sample size of 200 subjects over both Pre- and Post- Surgical Expansion Surveys. For the Pre-Surgical Expansion survey, the study team stopped data collection at 3 months, when the target of 100 participants was nearly reached. All eligible women were approached for recruitment during this time period.

### Data collection and processing

Bayalpata Hospital Pre-Surgical Expansion Survey data were used to examine attitudes and behaviors toward institutional birth before the establishment of the hospital’s comprehensive emergency obstetric care facility. The survey included demographic inquiries and questions to assess the decision-making process, accessibility of institutional birth, and acceptability of services. In addition, patient narratives of their birth stories and their recommendations for improving utilization of institutional services were collected. Community Health Worker Leaders (CHWLs), paid employees in Bayalpata Hospital’s Community Health Program managing the CHWs, administered the surveys to post-partum women in the catchment area population of the hospital who had given birth at home or at a village clinic in the prior 6 weeks. If more than one woman in a household was eligible for participation, all eligible women were approached for enrollment. Auxiliary Nurse Midwives (ANMs) administered surveys to post-partum women who had given birth at the hospital in the prior 6 weeks, inclusive of both uncomplicated and complicated births. An attempt was made to reach all post-partum women during the study period. Surveys were printed in Nepali and enumerators conducted the surveys in Nepali or in Achhami, the local dialect. *Possible’s* Director of Research was present for the majority of survey administration to ensure consistency. The CHWs and ANMs were also given weekly reminders to complete the survey forms.

### Outcome measures

The primary outcome was location of birth. The goals were to describe and quantify factors associated with institutional birth and home birth, and to understand which factors are significant predictors of institutional birth. The following factors were examined: age, distance from hospital, household income, land ownership, race, literacy, parity, antenatal care, birth decision-making, and father’s presence. The qualitative analysis was based on the social contextual theoretical framework [15], and aimed to identify modifying and mediating factors in relation to the future intervention of comprehensive emergency obstetric care services.

### Quantitative analysis

All data were collected on paper forms and input into Excel (Microsoft Corp., Richmond WA) by the staff of the Community Health Program and the Hospital Program at Bayalpata Hospital. For this study, survey data were analyzed using Stata 12.1 (Stata Corporation, College Station, TX) and JMP Pro version 10.0.0. Data collection tools were pilot tested for errors at Bayalpata Hospital prior to the survey.

Each variable was examined by location of birth. For categorical variables, cell counts were examined. Each continuous variable was examined for normality of distribution. For normally distributed variables, means and standard deviations were calculated, for non-normal data, medians and inter-quartile ranges were calculated.

For the logistic regression, a manual forward-selection method was used, starting with scientifically important covariates: age, parity, literacy, income, and distance from the hospital. A covariate in the model was kept if it was significant (*p* < 0.10) or if it altered the beta coefficient by more than 20 %. This process led to a final logistic regression model with five covariates: parity, literacy, monthly household income, land, and presence of father of baby. To examine the effect of these covariates on the outcome of location of birth, odds ratios with 95 % confidence intervals were used. To test the significance of the effects, the likelihood ratio tests were used.

In addition to the regression analysis, descriptive statistics of perceptions of care, accessibility and acceptability of services, and major recommendations for improving institutional birth utilization were examined.

### Qualitative analysis

A single open-ended question was posed at the start of each survey: “Tell me the story of your birth.” Responses were transcribed on the survey by the interviewer in Nepali. These responses were then translated into English for analysis. Analysis was based on the social contextual model [15]. This theoretical framework helped to illuminate pathways by which social contextual factors lead to differing health outcomes or health behaviors. Based on the model, these factors were then examined as they may or may not be affected by a proposed intervention.

For this study, the outcome of interest was institutional birth and the proposed intervention was expansion of services at Bayalpata Hospital from basic to comprehensive emergency obstetric care, specifically providing blood banking and cesarean sections. Through immersion crystallization by two investigators, modifying and mediating factors were identified [16]. Modifying factors were thought to independently affect the outcome, and not be influenced by the intervention. Mediating factors were thought to be on the pathway between the intervention and the outcome. The two investigators identified and categorized these factors prior to the roll out of the intervention using data from Pre-Surgical Expansion Survey. These factors occurred on multiple levels: individual, interpersonal, organizational, community, and societal.

## Results

Over a 3-month collection period in 2012, 98 subjects were enrolled in the Pre-Surgical Expansion Assessment study. Of these, 57 of the women gave birth at home and 39 gave birth at a health institution. Of the final total of enrolled women, 77 were from the catchment area population; the remaining 21 women were from neighboring communities outside the catchment area population, but who received healthcare services at Bayalpata Hospital. The reported institutional birth rate presented is restricted to the 77 women from the catchment area population, as an accurate rate could not be calculated to include neighboring communities. The remainder of the analysis included all 98 women in order to maximize power in looking at predictors of institutional birth.

The distributions of characteristics by location of birth are presented in Table 1. Mean participant age and median distance from the hospital were similar between groups. For the institutional birth group, the median monthly household income was Nepali Rupees (NRs) 5000 (IQR 3000, 10000) and that for the home birth group was NRs 200 (IQR 0, 3250). Median monthly household income was much greater in the institutional birth group, however median amount of land was greater in the home birth group. Forty-six percent of home births were from the Dalit caste (a disadvantaged socio-ethnic group stratified by the Hindu caste system), versus only 37 % of institutional births. Seventy seven percent of home births were of multiparous women, compared to 61 % of institutional births. Percent of adequate antenatal care and self-birth-decision-making were similar between groups. In 76 % of institutional births, the father of the baby was present in the district at the time of post-partum interview, compared with just 53 % of home births.

**Table 1:** Characteristics of the study samples: Comparison of institutional versus home births

Characteristic	Institutional Birth (*n* = 39, 40 %)	Home Birth (*n* = 57, 60 %)	*P*-Value
Age, years, mean (SD)	24 (6)	25 (4)	0.61
Distance from district hospital, hours, median (IQR)	2 (1, 2.9)	2 (1.5, 2)	0.33
Monthly household income, Nepali Rupees, median (IQR)	5000 (3000, 10000)	200 (0, 3250)	0.02
Land, Ropani^a^, median (IQR)	3 (1, 7.5)	5 (2, 12)	0.06
Caste, number (%)			
Dalit	15 (37)	26 (46)	0.37
Literacy, number (%)			
Literate	30 (73)	28 (49)	0.02
Parity, number (%)			
Primiparity	16 (39)	13 (23)	0.08
Antenatal care, number (%)			
Adequate^b^	30 (73)	39 (68)	0.61
Birth decision making, number (%)			
Self alone	14 (34)	17 (30)	0.69
Presence of father of baby, number (%)			
Present	31 (76)	30 (53)	0.02

^a^Ropani is a commonly used unit in Nepal to measure land and is equal to 508.72 m^2^

^b^Defined as at least 4 antenatal care visits, per Nepali Government guidelines

The logistic regression model (Table 2) explained 39 % of the variability in the data. Age, monthly household income, and land were significant predictors of institutional birth (*p* < 0.05). Because age and monthly household income have a non-linear relationship with institutional birth, squared terms were included in the final regression model. As age increased, the likelihood of institutional birth decreased. The odds ratio was higher in older age groups (Table 3). As income increased, the likelihood of an institutional birth also increased. However, beyond a certain point, additional income had less of an effect on birth location (Table 4). Income and land, both measures of socio-economic status, are expected to be related to birth location in the same direction. In the study data, they relate in opposite directions. A higher monthly household income predicts institutional birth, whereas a lower amount of land ownership predicts institutional birth.

**Table 2:** Results of logistic regression: likelihood of institutional birth by predictors

Covariate^a^	OR [95 % CI]	*P*-Value (likelihood ratio)
Age	0.06 [0.01, 0.33]	0.0008
Age^2	1.06 [1.03, 1.11]	0.0003
Land, Ropani	0.82 [0.71, 0.92]	0.0001
Monthly household income, in 1000 Nepali Rupees (NR)	1.45 [1.16, 1.88]	0.0006
Monthly household income^2, in 1000 NR	0.99 [0.98, 0.99]	0.0021
Parity	0.69 [0.13, 3.89]	0.6640
Literacy	0.33 [0.07, 1.40]	0.1323
Presence of father of baby	0.40 [0.09, 1.73]	0.2215
Distance	1.09 [0.67, 1.73]	0.7218

^a^Reference categories: for Parity, Nulliparous; for Literacy, Illiterate; and for Presence of father of baby, Absent

**Table 3:** Odds Ratios for adjusted age as a predictor of institutional birth

Percentile	Age	OR
25	21	0.20
50	24	0.23
75	27.25	0.28

**Table 4:** Odds Ratios for adjusted monthly household income as a predictor of institutional birth

Percentile	Monthly household income, in 1000 NR	OR
25	0.0075	1.45
50	2.25	1.42
75	5.25	1.38

**Fig. 1:**
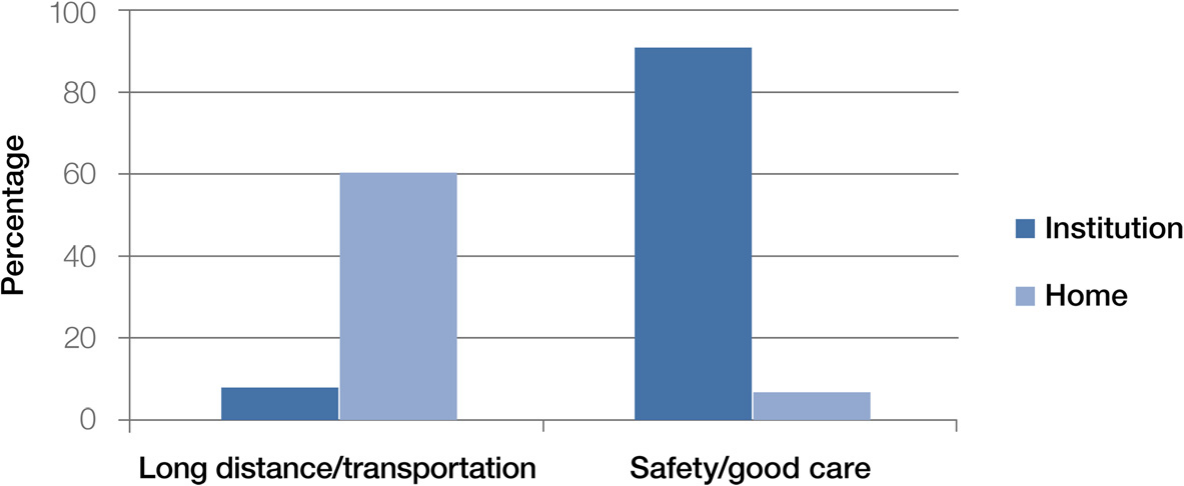
Location of birth and most important factors

When asked about the most important factor in determining the location of birth, most women in the institutional birth group selected "safety/good care" (90 %), whereas most women in the home birth group selected "long distance" (60 %, Fig. 1). Giving birth at the hospital was considered to be safer by the majority of both the institutional birth group (98 %) and the home birth group (88 %).

The cost of travel to the birth location for the institutional birth group was NRs 831 (CI 486.5–1174.6). Overall, the most common participant recommendation for improving institutional service utilization was improving ambulance accessibility (72 %); only 37 % of the institutional birth sample arrived via an ambulance.

In terms of acceptability of services, institutional birth satisfaction was at 93 % while home birth satisfaction was 32 %. All participants in the institutional birth group stated they would prefer an institutional birth in the future, while only one participant from the home birth group stated a preference for home birth in the future.

**Fig. 2:**
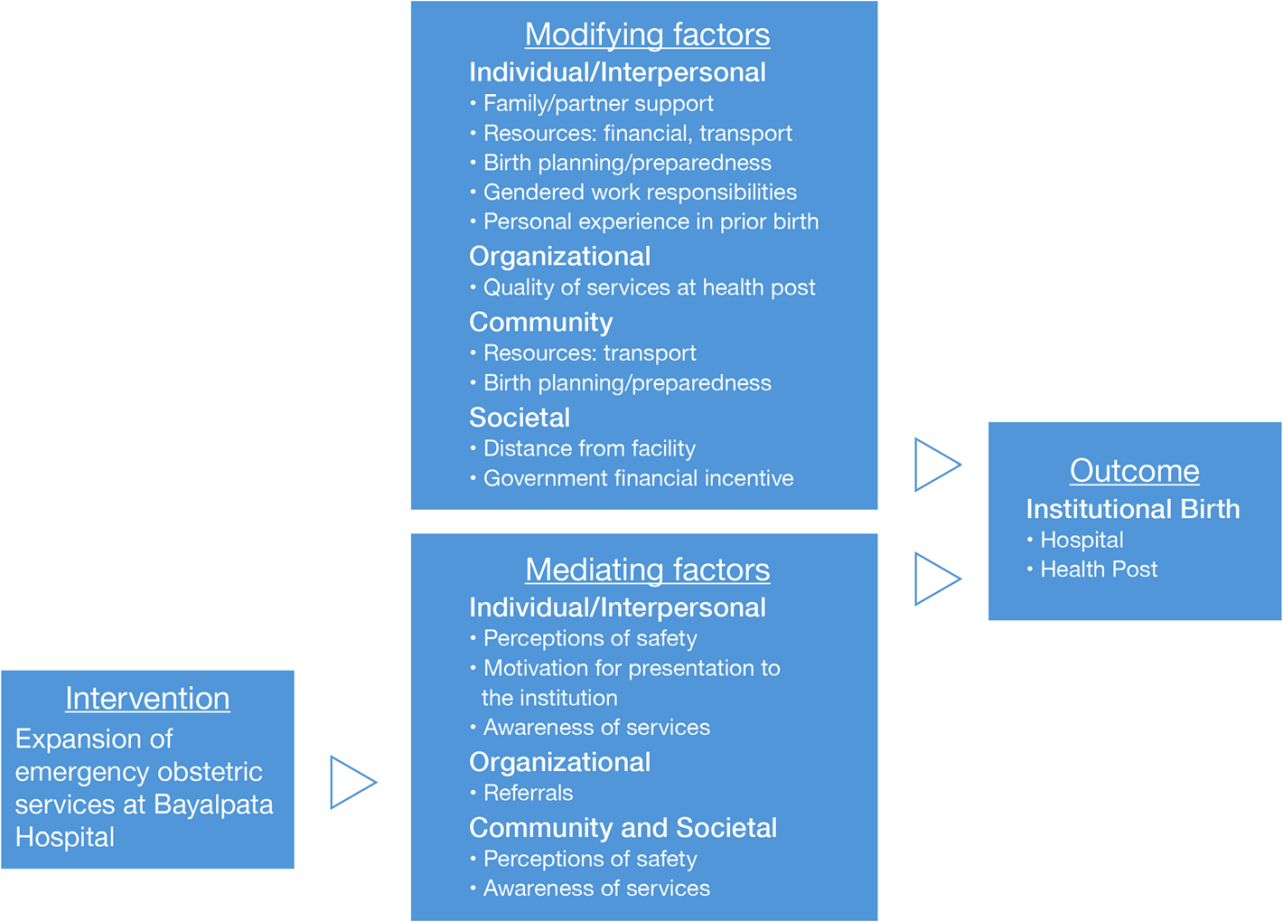
Factors affecting institutional birth. *Note: The mediating factors were defined as likely being affected by the proposed intervention while the modifying were likely not going to be affected by the intervention

The qualitative analysis examined women’s ‘birth stories’ for social contextual factors as they would relate to the proposed expansion of emergency obstetric services. Modifying factors and mediating factors were identified and fit into a conceptual model (Fig. 2). Modifying factors at the individual and interpersonal level included family (particularly mother-in-law) and partner support, access to financial resources and means of transport to an institutional setting, birth planning and preparedness, gendered work responsibilities, and positive or negative personal experiences during a prior birth.

The importance of family and partner support was identified in the stories of many women, as this quote exemplifies: “She wanted to deliver in the hospital but her in-laws did not listen. Her husband was not in Achham. She didn't have the courage to come to the hospital by herself so she delivered at home.” The factors of access to transportation, birth planning, and preparedness were also seen as community level factors due to the need for several community members to participate in the process of getting to an institution: “A [20–30] year old woman who was pregnant with [a] child was herding her goats during the day. She went into labor around 7 pm. The family was trying to gather people to bring her to the hospital but she had her baby an hour and half later.” A societal level modifying factor identified was the quality of services at the village clinics, which was often poor: “They wanted to go to the village clinic but no medical staff were there.” The government-provided financial incentive was another societal level modifying factor that motivated women to have an institutional birth.

The birth stories also revealed social and contextual factors that would likely be affected by the proposed expansion of services. On an individual and interpersonal level, perceptions of safety and intention to present to an institution seemed important for achieving institutional birth, as this woman’s story exemplifies:

“[A 40–50] year old woman who had [multiple] prior deliveries in her home, just had [a] delivery in the hospital. One year ago there was a maternal death in a nearby village while a woman was delivering at home. News of this death changed her mind about having her [next] baby at home. She wanted to come to the hospital, where there was a doctor and good medical staff to keep her safe.”

Unlike this story of a woman who achieved institutional birth, there were several women who gave birth at home and mentioned that they had limited awareness of the hospital or services provided there. Referral to a higher level of care was an organizational level mediating factor. Three women were referred to an institution with comprehensive emergency obstetric care services, but delivered at home due to delays caused by referral.

## Discussion

The results of this study are in line with prior literature, in that age, income, and land, were significantly related to institutional birth. However, age is inversely related to institutional birth, with younger women more likely to give birth in an institution. The direction of this relationship is contrary to findings in other settings [6]. This may be explained by circumstances in rural Nepal that are different than in other parts of the world, such as literacy. In this study, the average age of literate women is significantly younger (23) than that of illiterate women (27). Literacy may be a proxy for other characteristics, such as empowerment and independence, which could impact usage of institutional birth services [17].

Higher income predicted institutional birth in our analysis. The non-linear relationship, however, reveals that the amount of income matters less in the higher range for achieving institutional birth. Land has an opposite effect on institutional birth, as ownership of more land negatively predicts institutional birth. Perhaps this is due to the specific very rural context of the study as families with more land are unlikely to be earning other sources of cash revenue. In a remote area like Achham, cash is important to secure transportation. Perhaps those with less land had easier access to that cash. Further analysis of the current data, however, shows no significant association between land and income. This issue warrants further research in a larger sample of women or with qualitative methods.

Findings suggest that the most important consideration for women is safety/good care among the institutional birth group and long distance/transportation in the home birth group. The majority of all women stated that the hospital was safer place to give birth and would prefer an institutional birth in the future. These findings indicate that underutilization of institutional birth services is unlikely to be the result of patient preference, and rather due to socioeconomic vulnerability to structural barriers.

Although distance from the hospital was not a significant predictor of institutional birth in the final regression model, ‘long distance or transportation’ was the most important consideration reported by the home birth group. Other studies show distance to be a significant factor that affects choice of birthplace [18, 19]. In this study, the most common participant recommendation to increase institutional service utilization was to improve ambulance accessibility and currently only a minority of those who gave birth at an institution arrived via an ambulance. Thus, the delay in arriving at the hospital is an area that must be addressed if institutional birth rates are to improve. Improving transportation delays have been shown in other studies to reduce neonatal mortality and stillbirths, when integrated with community mobilization, education, and facility improvement [20].

The mediating social and contextual factors identified in the qualitative analysis are those that will most likely be impacted by the proposed expansion of emergency obstetric services: perceptions of safety, intent to give birth in the hospital, referrals, and awareness of services. On the other hand, modifying social and contextual factors, such as social support, gender equality, financial and transportation resources, and birth preparedness, are less likely to be impacted by the expansion of services. Thus, they should be the targets of interventions conducted alongside expansion of emergency obstetric services to improve institutional birth in this region.

Limitations to this study include a small sample size that was made smaller by missing values. The power calculation was for the larger study, comparing Pre- and Post- Surgical Expansion Survey data, not for the present analysis of determining factors associated with location of birth. Thus, the study may be under-powered to detect all factors associated with institutional birth. The convenience sampling strategy employed may have biased the findings. Future research with a consecutive sampling strategy should be employed to address this issue. Additionally, the institutional birth rate among this sample (42 %) was higher than the estimated rate in the hospital catchment area population (30 %). The skew towards women who had an institutional birth brings bias to the sample. The use of community health workers and nurse midwives as enumerators also increases bias in self-reported perceptions of and preferences for institutional birth. The generalizability of the findings is limited due to the data being obtained from a single hospital and its catchment area population. Finally, due to resource limitations in our setting, qualitative interviews could not be audio-recorded, perhaps leading to error in transcription and translation of participant responses. Despite these limitations, this study makes a unique contribution to the small and growing literature on determinants of institutional birth in the Global South.

## Conclusions

In exploring determinants of institutional birth in rural Nepal, we found that socioeconomic vulnerability to structural barriers are paramount in understanding who gives birth at home versus an institution and why. Contrary to data from the Nepal Demographic and Health Survey 2011, where 62 % of women who gave birth at home believed it was not necessary to give birth in a health facility [21], most women surveyed in our study, in rural Nepal in 2012, believed that it was safer to have an institutional birth. Despite this difference in thinking, the majority of women in our study still gave birth at home. Efforts to reduce maternal mortality must both make institutions safer by expanding to comprehensive emergency obstetric services, as well as target the underlying social and economic inequity and structural barriers that prevent women from achieving institutional birth.

## Additional file

Additional file 1: Institutional Birth Survey_Pre-Surgical Expansion_Eng,Nep (PDF 257 kb)

## Abbreviations

ANM(s): Auxiliary Nurse Midwives
CHW(s): Community Health Worker(s)
CHWL(s): Community Health Worker Leader(s)
IQR(s): Interquartile Range(s)
NRs: Nepali Rupees
OR(s): Odds Ratio(s)
SD: Standard Deviation
UN: United Nations
