# Supplementary material for: Determinants of institutional birth among women in rural Nepal: a mixed-methods cross-sectional study
Source: BMC Pregnancy Childbirth. 2016 Aug 27;16(1):252. doi: 10.1186/s12884-016-1022-9 (PMC5002328; doi:10.1186/s12884-016-1022-9)
Supplement: Additional file 1: — Institutional Birth Survey_Pre-Surgical Expansion_Eng,Nep (PDF 257 kb) [file 12884_2016_1022_MOESM1_ESM.pdf]

Determinants of institutional delivery: Female Survey

Study ID ______________________

INTERVIER NAME: ______________________

DEMOGRAPHIC INFORMATION

1. **How old are you?**
   ______years
2. **Where do you live?**
   District: ______________________
   VDC: ________________ **Ward:** ______
   Distance to Bayalpata Hospital: _____ **hours**
3. **What is your caste?**
   ☐ Dalit
   ☐ Brahmin
   ☐ Kshetrya
   ☐ Vaishya
   ☐ Any other caste
4. **How much money does the household make on average per month?** ______ NRs
5. **How many ropani land does your household own?**
   ___________ ropani
6. **What is the highest level of education you have completed? (*Please choose only one.*)**
   ☐ Illiterate
   ☐ Literate, no formal schooling
   ☐ Literate, schooling up to primary only
   ☐ Literate, schooling through secondary+
7. **How many times have you been pregnant, including abortion, miscarriage, cesarean section?**
   ______times
8. **How many vaginal deliveries have you had? (including most recent)**
   ______vaginal deliveries
9. **How many cesarean sections have you had?**
   ______cesarean sections
10. **How many living children?**
    ______sons ______daughters
11. **How many antenatal care visits in this pregnancy (0=none)?**
    _______visits
12. **If so, location of antenatal care:**
    ☐ Heath post
    ☐ Hospital
    ☐ Private clinic

DECIDING TO SEEK CARE

13. **Where did you deliver your baby?**
    ☐ Home/House/Friend's;VDC______________
    ☐ Health post; Name __________________
    ☐ Hospital; Name _____________________
    ☐ Private clinic; Name ________________
    ☐ Other: _____________
14. **Who made the decision about location of delivery?**
    ☐ Self
    ☐ Husband
    ☐ Other male relative/in-law/friend
    ☐ Other female relative/in-law/friend
    ☐ I don't know/Other________
15. **What were the most important factors in making this decision?** ***Do not give options; tick all that she mentions***
    ☐ Cost of care
    ☐ Safety
    ☐ Distance
    ☐ Availability of c-section/blood transfusion
    ☐ Treatment by medical staff
    ☐ Financial incentive
    ☐ Privacy
    ☐ Space
    ☐ I don't know/Other___________
16. **What symptoms or conditions prompted you to seek care (select all that apply)?**
    ☐ Contractions/Knew I was to deliver
    ☐ Bleeding
    ☐ Pain not related to contractions
    ☐ Previous complicated pregnancies
    ☐ Concern for my health
    ☐ Partner/spouse/family encouragement/suggestion
17. **Do you feel that it is safer to deliver at home or in the hospital?**
    ☐ Home
    ☐ Hospital
    ☐ Same
    ☐ I don't know/Other_________
18. **Are cesarean section services offered at Bayalpata Hospital?**
    ☐ Yes
    ☐ No
    ☐ I don't know/Other_________
19. **Are blood transfusion services offered at Bayalpata Hospital?**
    ☐ Yes
    ☐ No
    ☐ I don't know/Other_________

20. **Did you know prior to your delivery about whether cesarean section and blood transfusion services are offered at Bayalpata Hospital?**
    ☐ Yes
    ☐ No
    ☐ I don't know/Other_________
21. **If yes, how did you know?**
    ☐ Antenatal clinic
    ☐ Doctor/health care worker
    ☐ Word of mouth
    ☐ Community health worker
    ☐ Radio announcement
    ☐ I don't know/Other_________
22. **If yes, did this affect your decision on where to deliver?**
    ☐ Yes
    ☐ No
23. **Where do you plan to deliver in the future if you become pregnant again?**
    ☐ Home
    ☐ Health post
    ☐ Hospital
    ☐ Private clinic
    ☐ I don't know/Other_________

ACCESSING CARE

24. **Where were you immediately prior to delivery?**
    ☐ Home
    ☐ Health post
    ☐ Hospital
    ☐ Private clinic
    ☐ I don't know/Other_________
25. **During your pregnancy, did you change your home location in preparation for delivery?**
    ☐ Yes; Distance from home: _______ hours;
    Location name: ________________________
    Why?__________________________________
    ☐ No
26. **How long did it take to get to where you delivered?**
    ______ Hours
27. **What did it cost to travel to where you delivered?**
    ___________/Rs.
28. **How much did you pay to deliver your baby?**
    _____________/Rs.
29. **Did you/will you receive the government financial incentive for delivering your baby where you did?**
    ☐ Yes
    ☐ No
    ☐ I don't know/Other_________
30. **Was travel a factor for where you delivered?**
    ☐ Major Barrier
    ☐ Minor Barrier
    ☐ No Barrier
    ☐ I don't know/Other
31. **Was high cost factor for where you delivered?**
    ☐ Major Barrier
    ☐ Minor Barrier
    ☐ No Barrier
    ☐ I don't know/Other
32. **How can the hospital make it easier for you to arrive to the hospital and receive services?**
    ____
    ____

QUALITY OF CARE RECEIVED

33. **What do you think is required for a safe delivery?**
    ____
    ____
34. **Was lack of privacy factor for where you delivered?**
    ☐ Major Barrier
    ☐ Minor Barrier
    ☐ No Barrier
    ☐ I don't know/Other
35. **Was concern about treatment by medical staff a factor for where you delivered?**
    ☐ Major Barrier
    ☐ Minor Barrier
    ☐ No Barrier
    ☐ I don't know/Other
36. **What service did you receive at where you delivered (all that apply)?**
    ☐ Vaginal delivery
    ☐ Cesarean section
    ☐ Manual removal placenta
    ☐ Postpartum hemorrhage treatment
    ☐ Suture of laceration
    ☐ Blood transfusion
    ☐ Treatment for eclampsia
    ☐ I don't know/Other ________
37. **Did where you deliver have adequate staff to provide you a safe delivery?**
    ☐ Completely adequate
    ☐ Somewhat adequate, why?_____________

☐ Not adequate, why? ________________
☐ I don't know/Other________

38. Did where you deliver have adequate supplies to provide you a safe delivery?
☐ Completely adequate
☐ Somewhat adequate, why? ______________
☐ Not adequate, why? _________________
☐ I don't know/Other________

39. Did where you deliver have adequate space to provide you a safe delivery?
☐ Completely adequate
☐ Somewhat adequate, why? __________________
☐ Not adequate, why? ______________________
☐ I don't know/Other________

40. What was lacking at the place of delivery during your delivery?
____
____

41. Were you satisfied with the care that you received for your delivery?
☐ Completely satisfied
☐ Mostly satisfied, why?________________________
☐ Mostly dissatisfied, why?________________________
☐ Completely dissatisfied, why?_____________________
☐ I don't know/Other_________________________

42. Were you satisfied with immediate post-partum care you received?
☐ Completely satisfied
☐ Mostly satisfied, why?________________________
☐ Mostly dissatisfied, why?________________________
☐ Completely dissatisfied, why?_____________________
☐ I don't know/Other_________________________

POSTPARTUM CONTRACEPTION

43. Did you/will you receive a method of family planning or contraception after delivery
☐ Yes
☐ No
☐ I don't know

44. If so, what method?
☐ Condoms
☐ Depo
☐ IUD
☐ Norplant
☐ Tubal ligation
☐ OCP/Pills
☐ None/I don't know/ Other: ___________

MALE FAMILY MEMBER SURVEY

45. Where is the father of your infant?
☐ Working; in Achham
☐ Working; not in Achham
☐ Separated
☐ Not living
☐ I don't know/Other____________

46. Do you feel comfortable letting us speak to a male decision maker in your household who is present today to ask them some similar questions?
☐ Yes
☐ No

THANK YOU!
☐ NRs. 100 given

Determinants of institutional delivery: Male Survey

Linked Study ID ______________________

INTERVIER NAME: ______________________

DEMOGRAPHIC INFORMATION

1. **What is your relationship to the woman who delivered?**
   ☐ Husband/Partner
   ☐ Father
   ☐ Father-in-law
   ☐ Other male relative
   ☐ Other male-in-law
   ☐ Non-related/non-in-law friend
2. **How old are you?**
   ______years
3. **What is your caste?**
   ☐ Dalit
   ☐ Brahmin
   ☐ Kshetrya
   ☐ Vaishya
   ☐ Any other caste
4. **What is the highest level of education you have completed? (*Please choose only one.*)**
   ☐ Illiterate
   ☐ Literate, no formal schooling
   ☐ Literate, schooling up to primary only
   ☐ Literate, schooling through secondary+

DECIDING TO SEEK CARE

5. **Who made the decision for her to deliver at that location?**
   ☐ Woman herself
   ☐ Husband
   ☐ Other male relative/in-law/friend
   ☐ Other female relative/in-law/friend
   ☐ I don't know/Other________
6. **What were the most important factors in making this decision?** ***Do not give options; tick all that she mentions***
   ☐ Cost of care
   ☐ Safety
   ☐ Distance
   ☐ Availability of c-section/blood transfusion
   ☐ Treatment by medical staff
   ☐ Financial incentive
   ☐ Privacy
   ☐ Space
   ☐ I don't know/Other___________
7. **Do you feel that it is safer to deliver at home or in the hospital?**
   ☐ Home
   ☐ Hospital
   ☐ Same
   ☐ I don't know/Other_________
8. **Did you know prior to her delivery about whether cesarean section and blood transfusion services are offered at Bayalpata Hospital?**
   ☐ Yes
   ☐ No
   ☐ I don't know/Other_________
9. **If yes, how did you know?**
   ☐ Antenatal clinic
   ☐ Doctor/health care worker
   ☐ Word of mouth
   ☐ Community health worker
   ☐ Radio announcement
   ☐ I don't know/Other_________
10. **If yes, did this affect the decision on where to deliver?**
    ☐ Yes
    ☐ No
11. **Are cesarean section services offered at Bayalpata Hospital?**
    ☐ Yes
    ☐ No
    ☐ I don't know/Other_________
12. **Are blood transfusion services offered at Bayalpata Hospital?**
    ☐ Yes
    ☐ No
    ☐ I don't know/Other_________
13. **Where do you think she should deliver in the future if she becomes pregnant again?**
    ☐ Home
    ☐ Health post
    ☐ Hospital
    ☐ Private clinic
    ☐ I don't know/Other_________

ACCESSING CARE

14. **Was travel a factor for where she delivered?**
    ☐ Major Barrier
    ☐ Minor Barrier
    ☐ No Barrier
    ☐ I don't know/Other

15. Was high cost a factor for where she delivered?

☐ Major Barrier
☐ Minor Barrier
☐ No Barrier
☐ I don't know/Other

16. **How can the hospital make it easier for her to arrive to the Hospital and receive services?**

____
____

QUALITY OF CARE RECEIVED

17. What do you think is required for a safe delivery?

____
____

18. Was lack of privacy a factor for where she delivered?

☐ Major Barrier
☐ Minor Barrier
☐ No Barrier
☐ I don't know/Other

19. Was concern about treatment by medical staff a factor for where she delivered?

☐ Major Barrier
☐ Minor Barrier
☐ No Barrier
☐ I don't know/Other

20. Did where she delivered have adequate staff to provide her a safe delivery?

☐ Completely adequate
☐ Somewhat adequate, why?______________
☐ Not adequate, why? ___________________
☐ I don't know/Other_________

21. Did where she delivered have adequate supplies to provide you a safe delivery?

☐ Completely adequate
☐ Somewhat adequate, why? _______________
☐ Not adequate, why? ____________________
☐ I don't know/Other_________

22. Did where she delivered have adequate space to provide you a safe delivery?

☐ Completely adequate
☐ Somewhat adequate, why? ____________________
☐ Not adequate, why? _________________________
☐ I don't know/Other_________

23. What was lacking at the place of delivery during her delivery?

____
____

24. Were you satisfied with the care that you received for her delivery?

☐ Completely satisfied
☐ Mostly satisfied, why?__________________________
☐ Mostly dissatisfied, why?___________________________
☐ Completely dissatisfied, why?_______________________
☐ I don't know/Other____________________________

25. Were you satisfied with immediate post-partum care she received?

☐ Completely satisfied
☐ Mostly satisfied, why?__________________________
☐ Mostly dissatisfied, why?___________________________
☐ Completely dissatisfied, why?_______________________
☐ I don't know/Other____________________________

THANK YOU FOR YOUR PARTICIPATION!

मिति

अन्तरवार्ता लिनेको नाम: ________________________

तपाईले बच्चा जन्माउदा खेरीको कथा भन्नुहोस् । के कस्ता योजनाहरु गर्नुभएको थियो, के भयो र कस्तो महसुस गर्नुभयो ।

जनसांख्यकी सूचना

1. **तपाई कति वर्षको हुनु भयो?**
   ______ वर्ष
2. **तपाई कहाँ बस्नु हुन्छ?**
   जिल्ला : ______________________________
   गा.वि.स.: ______________________ वडा न.: _____
   तपाइको घर देखी वयालपाटा अस्पताल आउन कति समय लाग्छ ?_____ घण्टा
3. **तपाई को जात के हो ?**
   ☐ दलीत
   ☐ व्रामण
   ☐ क्षेत्री
   ☐ वैश्य
   ☐ अन्य
4. **तपाईको पविारले महिनामा कति आम्दानी गर्नुहुन्छ ?**
   रु ______
5. **तपाई संग जम्मा कती रोपनि जमीन छ ?**
   __________ रोपनि
6. **तपाईले कति पढ्नु भएको छ ? (कुनै एक उत्तर छान्नुहोस्)**
   ☐ अशिक्षीत
   ☐ साधारण लेखपढ
   ☐ शिक्षीत, प्रा.वि.सम्म
   ☐ शिक्षीत,मा.वि+
7. **गर्भपात,गर्भपतन तथा अन्य लगायत तपाई कती पटक गर्भवति हुनु भएको छ ?**
   ______ पटक
8. **तपाईले कति बच्चा घरैमा जन्माउनु भयो ?**
   ______ पटक
9. **तपाईले अस्पतालमा कती बच्चा जन्माउनुभयो ?**
   ______ पटक
10. **अपरेशन गरेर तपाईले कति पटक सुत्केरी गराउनु भयो ?**
    ______ पटक
11. **तपाईका कती वटा बच्चाहरु छन् ?**
    ______छोरा ______छोरी
12. **गर्भवति अवस्थामा तपाईले कती पटक चेक जाँच गराउनु भयो ?**
    _______ पटक

    (यदि '०' उत्तर आएमा प्रश्न नं.१४ मा जानु)
13. **यदि चेक जाँच गराउनु भयो भने तलका मध्ये कहाँ गराउनु भयो ?**
    ☐ स्वास्थ्य चौकी
    ☐ अस्पताल
    ☐ निजी क्लिनिक

DECIDING TO SEEK CARE

14. **तपाई ले अन्तिम बच्चा कहाँ पाउनु भयो ?**
    ☐ घर/नातेदारको/साथीको घर, गा.वि.स.__________
    ☐ स्वास्थ चौकी; नाम ________________
    ☐ अस्पताल; नाम ____________________
    ☐ निजी क्लिनिक; नाम _______________
    ☐ अन्य: ____________
15. **तपाईले अन्तिम बच्चा कहाँ पाउने भनने निर्णय कसले गरेको थियो ?**
    ☐ आफै
    ☐ श्रीमान्
    ☐ ससुराबा/ अन्य पुरुष नातेदार/ साथी
    ☐ सासुआमा/ अन्य महिला नातेदार/ साथी
    ☐ पूरै परिवार
    ☐ खै/अन्य ________
16. **माथिको निर्णय लिनमा तलमध्येका कुन तत्वले महत्वपुर्ण भुमिका खेलेको थियो ? (एक भन्दा बढी उत्तरमा टिक लगाउन मिल्छ)**
    ☐ उपचार मुल्य
    ☐ सुरक्षा /राम्रो सेवा
    ☐ दुरी/सवारी साधन
    ☐ शल्यक्रिया तथा रगत दिने सुविधा
    ☐ स्वास्थयकर्मीको कमी
    ☐ आर्थिक सुविधा
    ☐ गोपनियता/ठाउ
    ☐ खै/ अन्य __________
17. तपाईले गर्भ वा सुत्केरी हुने बेलामा केहि समस्या परेर डाक्टर कहाँ जानु भएको थियो ? *(यदि थिएन भने प्र.नं. १८ मा जानु)* **तपाई डाक्टर कहाँ के भएपछि जानुभयो (मिल्ने जति उत्तरमा ठिक चिन्ह लगाउनुहोस्)**
    ☐ तल्लो पेटको दुखाइले
    ☐ रगत बगीरहेकोले

☐ तल्लो पेटबाहेक अन्य दुखाइले
☐ अघिल्लो पटक सुत्केरी हुँदा गाऱ्हो भएकोले
☐ आफ्नो स्वास्थ्यलाई ख्याल गरेर
☐ लामो ब्यथा
☐ अरुको सल्लाहा सुझाव
☐ अन्य

18. तलका मध्ये तपाई सुत्केरी हुन कहाँ सुरक्षीत ठान्नुहुन्छ ?
☐ अस्पतालमा
☐ अन्य__________________
☐थाहा छनै

19. के तपाइलाई थाहा छ वयालपाटा अस्पतालमा शल्यक्रीयाको सुविधा छ भनेर ?
☐ छ
☐ छैन
☐ खैाअन्य _______

20. के तपाइलाई थाहा छ वयालपाटा अस्पतालमा रगत दिने सुविधा छ भनेर ?
☐ छ
☐ छैन
☐ थाहछैन ________

(यदि घरमा अन्तिम बच्चा जन्माएको भए प्रश्न नं. २२ मा जानु)

21. प्रसुती हुनु पूर्व के तपाईलाई वयालपाटा अस्पतालमा शल्यक्रिया तथा रगत दिने सुविधा छ की छैन भनेर थाहा थियो
☐ थियो
☐ थिएन ________ प्रश्न नं २२ मा जानुहोस्।
☐ खै/अन्य ________ प्रश्न नं २२ मा जानुहोस्।

a. यदि थाहा थियो भने कहाँ वाट थाहा पाउनुभयो ?
☐ गर्भजाँच
☐ डाक्टर/स्वास्थ्य सेवक
☐ अरु कसैले भनेर
☐ सामुदायिक स्वास्थ्य कार्यकर्ता
☐ रेडीयोवाट
☐ खै/ अन्य ________

b. यदि थाहा थियो भने प्रसुती कहाँ गर्ने निर्णय गर्दा त्यसले फरक पाऱ्यो ?
☐ पाऱ्यो
☐ पारेन

22. यदि तपाई फेरी गर्भवति हुनु भयो भने तपाई प्रसुती कहाँ हुने चाहाना राख्नुहुन्छ ?
☐ घरमा
☐ स्वास्थ्य चौकी
☐ अस्पताल
☐ निजी क्लिनिक
☐ खैाअन्य ________

ACCESSING CARE

23. तपाई सुत्केरी हुनु अगाडि कहाँ हुनुहुन्थ्यो?
☐ घरमा
☐ स्वास्थ्य चौकी
☐ अस्पतालमा
☐ निजी क्लिनिकमा
☐ खै/थाँहा छैन ________

24. तपाईले सुत्केरी गराउन सजिलो होस् भनेर बस्ने ठाँउ सर्नुभएको थियो ?
☐ थिए
घर वाट दुरी : ______ घण्टा, ______
ठाउको नाम : ____________________
बस्ने ठाँउ किन **परिवर्तन गर्नु भयो ?**________________
☐ थिएन

25. सुत्केरी गराउने ठाउ सम्म पुग्न तपाईलाई कति घण्टा लाग्यो ?
______ **घण्टा** *(घरमै जन्माएको भए ० लेख्ने)*

(यदि घरमा अन्तिम बच्चा जन्माएको भए प्रश्न नं. ३० मा जानु)

26. तपाइ प्रसुती हुने ठाउ सम्म कसरी पुग्नु भयो ?
☐ हिडेर
☐ सवारी साधन वाट
☐ एम्बुलेन्स वाट
☐ अन्य

(यदि 'हिडेर वा अन्य' भन्ने उत्तर आएमा प्रश्न नं.२८ मा जानु)

27. प्रसुती हुने ठाउ जान कति रुपैया लाग्यो ?
रु _______

28. तपाइले प्रसुती भए वाफत कति पैसा तिर्नू भयो ?
रु____________

29. तपाइ प्रसुती भएपछी सरकारी प्रोत्साहन रकम पाउनु भयो ?
☐पाए
☐ पाएन
☐ खै / थाहा छैन ________

30. तपाइ प्रसुती कहाँ हुने निर्णय लिनको लागी यातायात बाधक भएको थियो ?
☐ मुख्य बाधक
☐ सामान्य बाधक
☐ बाधक थिएन
☐ खैाथहा छैन

31. तपाइ प्रसुती कहाँ हुने निर्णय लिनको लागी पैसा बाधक भएको थियो
☐ मुख्य बाधक
☐ सामान्य बाधक
☐ बाधक थिएन

☐ खै।थहा छैन

32. अस्पताल आइ सेवा लीनको लागी अस्पतालले कसरी सजिलो पार्न सकिब्छ ?

☐ एम्बुलेन्सको सेवा अफ्र बढी राम्रो बनाउने।

☐ एक्बुलेन्सको शुल्क घटाउने।

☐ अस्पतालको कर्मचारीलाइ सुलभ रुपले कुरा गर्न पाउनु परयो।

☐ स्वयम सेविका संगै आउनु परयो ।

☐ अन्य ________________________

QUALITY OF CARE RECEIVED

33. तपाइलाई सुरक्षीत प्रसुतीसेवा को लागी कुन कुरा आवश्यक जस्तो लाग्छ ।

____

____

34. तपाइ प्रसुती कहाँ हुने निर्णय लिनको लागी गोपनियता एउटा कारण थियो ।

☐ मुख्य बाधक

☐ सामान्य बाधक

☐ बाधक थिएन

☐ खै।थहा छैन

35. तपाइ प्रसुती कहाँ हुने निर्णय लिनको लागी सेवा दिन डाक्टर छ की छैन भनेर बाधक भएको थियो

☐ मुख्य बाधक

☐ सामान्य बाधक

☐ बाधक थिएन

☐ खै।थहा छैन

(यदि घरमा अन्तिम बच्चा जन्माएको भए प्रश्न नं. ३८ मा जानु)

36. **तपाइ प्रसुती भएको ठाउमा के कस्ता सेवा पाउनु भयो ।**

☐ साधारण प्रसुती

☐ शल्यक्रिया प्रसुती

☐ हात हालेर साल निकाल्ने

☐ सुत्केरी अवस्थामा हुने रक्तश्रापको उपचार

☐ च्यातीएको ठाउमा सिलाउने

☐ रगत दिने

☐ कम्पनको उचार

☐ खै।अन्य _______

37. तपाइलाइ सुरक्षीत प्रसुती सेवा दिनको लागी तपाइ प्रसुती भएको ठाउमा प्रयाप्त कर्मचारी थिए ।

☐ प्रयाप्त

☐ प्रयाप्त थिएन किन_______________

☐ खै।अन्य _______

38. तपाइलाइ सुरक्षीत प्रसुती सेवा दिनको लागी तपाइ प्रसुती भएको ठाउमा प्रयाप्त आवश्यक सामाग्री थिए ।

☐ प्रयाप्त

☐ प्रयाप्त थिएन किन ______________

☐ खै।अन्य ________

39. तपाइलाइ सुरक्षीत प्रसुती सेवा दिनको लागी तपाइ प्रसुती भएको ठाउमा प्रयाप्त आवश्यक ठाउ थियो

☐ प्रयाप्त

☐ प्रयाप्त थिएन किन ______________

☐ खै।अन्य ________

40. तपाइ प्रसुती भएको वेला तपाइ लाइ केही कुराको कमी थियो जस्तो लाग्छ ।

☐ गोपनियताको कमी

☐ दक्ष जनशक्ति को कमी(डाक्टर,नर्स)

☐ सफा ठाउ थिएन

☐ आवश्यक खाट थिएन।

☐ अन्य ___________________________

☐ केही पनि नाई

41. **तपाइले पाएको प्रसुती सेवामा के तपाइ सन्तुष्टी हुनुहुन्छ ।**

☐ सन्तुष्टी

☐ असन्तुष्टी किन __________________

☐ खै।अन्य ______________________

(यदि घरमा अन्तिम बच्चा जन्माएको भए प्रश्न नं. ४३ मा जानु)

42. तपाइले पाएको तुरुन्त सुत्केरी सेवामा के तपाइ सन्तुष्टी हुनुहुन्छ ।

☐ सन्तुष्टी

☐ असन्तुष्टी किन _________________

☐ खै।अन्य ______________________

POSTPARTUM CONTRACEPTION

43. सुत्केरी पश्चात तपाइले कुनै परिवार नियोजनका साधनहरु प्रयोग गर्नूभएको छ ।

☐ छ

☐ छैन

☐ खै

(यदि 'छैन वा खै' भन्ने उत्तर आएमा अन्तरवार्ता सकियो)

44. यदि प्रयोग गर्नु भएको छ भने तलका मध्ये कुन साधन प्रयोग गर्नुभएको छ ।

☐ कन्डम

☐ डिपो

☐ आयुडी

☐ नरपलान्ट

☐ मिनी ल्याप

☐ पिल्स

☐ अन्य : __________

धन्यवाद THANK YOU!

☐ NRs. 100 given

Date

अन्तरवार्ता लिनेको नाम INTERVIER NAME:

____

जनसांख्यकी सूचना DEMOGRAPHIC INFORMATION

1. **What is your relationship to the woman who delivered?**
   ☐ Husband/Partner
   ☐ Father
   ☐ Father-in-law
   ☐ Other male relative
   ☐ Other male-in-law
   ☐ Non-related/non-in-law friend
2. **तपाई कति वर्षको हुनु भयो? How old are you?**
   ______ वर्ष years
3. **तपाई को जात के हो । What is your caste?**
   ☐ दलीत Dalit
   ☐ ब्रामण Brahmin
   ☐ क्षत्री Kshetrya
   ☐ वैश्य Vaishya
   ☐ अन्य Any other caste
4. **तपाईले कति पढ्नु भएको छ ? What is the highest level of education you have completed? (*Please choose only one.*)**
   ☐ अशिक्षीत Illiterate
   ☐ साधारण लेखपढ Literate, no formal schooling
   ☐ शिक्षीत, प्रा.वि.सम्म Literate, schooling through primary
   ☐ शिक्षीत,मा.वि+ Literate, schooling through secondary+

DECIDING TO SEEK CARE

5. **तपाईले बच्चा कहाँ पाउने भनने निर्णय कसले गरेको थियो ? Who made the decision about location of delivery?**
   ☐ आफै Self
   ☐ श्रमिान Husband
   ☐ ससुराबा/साथी Other male relative/in-law/friend
   ☐ सासुआमा/साथी Other female relative/in-law/friend
   ☐ Whole family
   ☐ खै।अन्य I don't know/Other________
6. **माथिको निर्णय लिनमा तलमध्येका कुन तत्वले महत्वपुर्ण भुमिका खेलेको थियो ? Why did you deliver where you did? *Check all that apply***
   ☐ उपचार मुल्य High cost of care
   ☐ सुरक्षा Safety/Good care
   ☐ दुरी Long distance/Transportation
   ☐ शल्यक्रिया तथा रगत दिने सुविधा Availability of c-section/blood transfusion/other special services
   ☐ Lack of medical staff
   ☐ आर्थिक सुविधा Financial incentive
   ☐ गोपनियता/ठाउ Privacy/Space
   ☐ खै।अन्य I don't know/Other__________
7. **तलका मध्ये तपाई सुत्केरी हुन कहाँ सुरक्षीत ठान्नुहुन्छ । Do you feel that it is safer to deliver at home or in the hospital?**
   ☐ घरमा Home
   ☐ अस्पतालमा Hospital
   ☐ Same
   ☐ अन्य।थाहा छनै I don't know/Other___________________
8. **के तपाइलाइ थाहा छ वयालपाटा अस्पतालमा शल्यक्रीयाको सुविधा छ भनेर । Are cesarean section services offered at Bayalpata Hospital?**
   ☐ छ Yes
   ☐ छैन No
   ☐ खै।अन्य I don't know/Other_________
9. **के तपाइलाइ थाहा छ वयालपाटा अस्पतालमा रगत दिने सुविधा छ भनेर Are blood transfusion services offered at Bayalpata Hospital?**
   ☐ छ Yes
   ☐ छैन No
   ☐ थाहाछैन I don't know/Other_________
10. **प्रसुती हुनु पुर्व के तपाईलाइ वयालपाटा अस्पतालमा शल्यक्रिया तथा रगत दिने सुविधा छ की छैन भनेर थाहा थियो Did you know prior to your delivery about whether cesarean section and blood transfusion services are offered at Bayalpata Hospital?**
    ☐ थियो Yes
    ☐ थिएन No
    ☐ खै।अन्य I don't know/Other_________
    a. **यदि थाहा थियो भने कहाँ वाट थाहापाउनुभयो If yes, how did you know?**
    ☐ गर्भजाँच Antenatal clinic
    ☐ डाक्टर।स्वास्थ्य सेवक Doctor/health care worker
    ☐ अरु कसैले भनेर Word of mouth
    ☐ सामुदायीक स्वास्थ्य कार्यकर्ता Community health worker
    ☐ रेडीयोवाट Radio announcement
    ☐ खै।अन्य I don't know/Other_________
    b. **यदि थहा थियो भने प्रसुती कहाँ गर्ने निर्णय गर्दा त्यसले फरक पा-या If yes, did this affect your decision on where to deliver?**
    ☐ Yesपा-यो
    ☐ Noपारेन
11. **यदि तपाई फेरी गर्भवति हुनु भयो भने तपाई प्रसुती कहाँ हुने चाहाना राख्नुहुन्छ । Where do you plan to deliver in the future if you become pregnant again?**

☐ घरमा Home
☐ स्वास्थ्य चौकी Health post
☐ अस्पताल Hospital
☐ निजी क्लिनिक Private clinic
☐ खै।अन्य I don't know/Other________खै।अन्य

ACCESSING CARE

12. **तपाइ प्रसुती कहाँ हुने निर्णय लिनको लागी यातायात बाधक भएको थियो । Was travel a factor for where you delivered?**
☐ मुख्य बाधक Major Barrier
☐ सामान्य बाधक Minor Barrier
☐ बाधक थिएन No Barrier
☐ खै।थहा छैन I don't know/Other

13. **तपाइ प्रसुती कहाँ हुने निर्णय लिनको लागी पैसा बाधक भएको थियो Was high cost factor for where you delivered?**
☐ मुख्य बाधक Major Barrier
☐ सामान्य बाधक Minor Barrier
☐ बाधक थिएन No Barrier
☐ खै।थहा छैन I don't know/Other

14. **अस्पताल आइ सेवा लीनको लागी अस्पतालले कसरी सजिलो पार्न सक्छ । How can the hospital make it easier for you to arrive to the hospital and receive services?**
☐ एम्बुलेन्सको सेवा अभ्र बढी राम्रो बनाउने। Improve the ambulance service
☐ एक्बुलेन्सको शुल्क घटाउने Reduce the ambulance charge.
☐ अस्पतालको कर्मचारीलाइ सुलभ रुपले कुरा गर्न पाउनु परयो। स्वयम सेविका संगै आउनु परयो Have FCHVs accompany mother to the hospital.
☐ अन्य I don't know/others ____________________________

QUALITY OF CARE RECEIVED

15. **तपाइलाइ सुरक्षीत प्रसुतीसेवा को लागी कुन कुरा आवश्यक जस्तो लाग्छ । What do you think is required for a safe delivery?**
OPTIONS

16. **तपाइ प्रसुती कहाँ हुने निर्णय लिनको लागी गोपनियता एउटा कारण थियो Was lack of privacy factor for deciding where to deliver?**
☐ मुख्य बाधक Major Barrier
☐ सामान्य बाधक Minor Barrier
☐ बाधक थिएन No Barrier
☐ खै।थहा छैन I don't know/Other

17. **तपाइ प्रसुती कहाँ हुने निर्णय लिनको लागी सेवा दिन डाक्टर छ की छैन भनेर बाधक भएको थियो Was concern about treatment by medical staff a factor deciding where to deliver?**
☐ मुख्य बाधक Major Barrier
☐ सामान्य बाधक Minor Barrier
☐ बाधक थिएन No Barrier
☐ खै।थहा छैन I don't know/Other

18. **तपाइलाइ सुरक्षीत प्रसुती सेवा दिनको लागी तपाइ प्रसुती भएको ठाउमा प्रयाप्त कर्मचारी थिए । Did where you deliver have adequate staff to provide you a safe delivery?**
☐ प्रयाप्त Adequate
☐ प्रयाप्त थिएन किन Not adequate, why? ________________
☐ खै।अन्य I don't know/Other ________

19. **तपाइलाइ सुरक्षीत प्रसुती सेवा दिनको लागी तपाइ प्रसुती भएको ठाउमा प्रयाप्त आवश्यक सामाग्री थिए । Did where you deliver have adequate supplies to provide you a safe delivery?**
☐ प्रयाप्त Adequate
☐ प्रयाप्त थिएन किन Not adequate, why? ________________
☐ खै।अन्य I don't know/Other ________

20. **तपाइलाइ सुरक्षीत प्रसुती सेवा दिनको लागी तपाइ प्रसुती भएको ठाउमा प्रयाप्त आवश्यक ठाउ थियो Did where you deliver have adequate space to provide you a safe delivery?**
☐ प्रयाप्त Adequate
☐ प्रयाप्त थिएन किन Not adequate, why? ________________
☐ खै।अन्य I don't know/Other ________

21. **तपाइ प्रसुती भएको वेला तपाइ लाइ कही कुराको कमी थियो जस्तो लाग्छ । What was lacking at the place of delivery during your delivery?**
☐ गोपनियताको कमी Lack of privacy
☐ दक्ष जनशक्ति को कमी(डाक्टर,नर्स) Lack of skilled manpower
☐ सफा ठाउ थिएन Lack of clean space
☐ आवश्यक खाट थिएन। Lack of sufficient beds
☐ अन्य Others ______________________________
☐ Nothing

22. **तपाइले पाएको प्रसुती सेवामा के तपाइ सन्तुष्टी हुनुहुन्छ । Were you satisfied with the care that you received for your delivery?**
☐ सन्तुष्टी Satisfied
☐ असन्तुष्टी किन Dissatisfied, why? __________________
☐ खै।अन्य I don't know/Other ________________________

23. **तपाइले पाएको तुरुन्त सुत्केरी सेवामा के तपाइ सन्तुष्टी हुनुहुन्छ । Were you satisfied with immediate post-partum care you received?**
☐ सन्तुष्टी Satisfied
☐ असन्तुष्टी किन Dissatisfied, why? __________________
☐ खै।अन्य I don't know/Other ________________________

THANK YOU FOR YOUR PARTICIPATION!
